# Correlates of suicidal ideation related to the COVID-19 Pandemic: Repeated cross-sectional nationally representative Canadian data

**DOI:** 10.1016/j.ssmph.2021.100988

**Published:** 2021-12-09

**Authors:** Corey McAuliffe, Javiera Pumarino, Kimberly C. Thomson, Chris Richardson, Allie Slemon, Travis Salway, Emily K. Jenkins

**Affiliations:** a School of Nursing, University of British Columbia, T201-2211 Westbrook Mall, Vancouver, British Columbia, V6T 2B5, Canada; b School of Population and Public Health, University of British Columbia, 2206 E Mall, Vancouver, British Columbia, V6T 1Z3, Canada; c Centre for Health Evaluation and Outcome Sciences, St. Paul's Hospital, 5881-1081 Burrard Street, Vancouver, British Columbia, V6Z 1Y6, Canada; d The Human Early Learning Partnership, University of British Columbia, Suite 440, 2206 East Mall, Vancouver, British Columbia, V6T 1Z3, Canada; e Faculty of Health Sciences, Simon Fraser University, 8888 University Dr., Burnaby, V5A1S6, Canada; f BC Centre for Disease Control, 655 West 12th Ave., Vancouver, BC, V5L 4R4, Canada; g Centre for Gender and Sexual Health Equity, 1190 Hornby St. (11th Floor), Vancouver, BC, V6Z 1Y6, Canada

**Keywords:** COVID-19 pandemic, Suicidal ideation, Suicidal thoughts, Mental health, Sociodemographic data, Inequities

## Abstract

**Objective:**

With significant levels of mental distress reported by populations, globally, the magnitude of suicidal ideation during and beyond the COVID-19 pandemic is a central concern. The goal of this study was to quantify the extent of pandemic-related suicidal ideation in the Canadian population during the first ten months of the pandemic and identify sociodemographic and pandemic-related stressors associated with increased risk of ideation.

**Method:**

Data were derived from three rounds of a mental health monitoring survey, nationally representative by age, gender, household income, and region, delivered online in May 2020, September 2020, and January 2021. Bivariate analyses were used to quantify the proportion of respondents in Canada reporting suicidal ideation by sociodemographic factors and pandemic-related stressors. Unadjusted and adjusted multivariable logistic regression was used to study the association between suicidal ideation and correlates within four pandemic-related stressor categories (financial, relationship, substance use, COVID-19 exposure).

**Results:**

Of the 7002 respondents, 6.2% (n = 433) reported experiencing suicidal thoughts or feelings as a result of the pandemic within the two weeks prior to taking the survey. In terms of sociodemographic factors, suicidal ideation was more commonly reported among those who were not cisgender, <65 years-old, single, Indigenous, LGBT2Q+, and who experience a pre-existing mental health condition. After adjusting for sociodemographic factors, indicators across all four pandemic-related stressor categories were associated with two or more times the odds of suicidal ideation.

**Conclusion:**

Disparities in COVID-19 related suicidal ideation have persisted throughout the first year of the pandemic for specific sociodemographic sub-groups and those who have faced stressors related to finances, relationships, increased substance use, and COVID-19 virus exposure. To best address these disparities and to prevent a transition from suicidal ideation to action, appropriate planning, resources, and policies are needed to ensure health and well-being for everyone.

## Highlights

• 6.7% of respondents reported experiencing suicidal thoughts/feelings due to the pandemic.
• Proportion of Canadians experiencing suicidal thoughts/feelings has increased.
• Sociodemographic characteristics associated with higher odds of suicidal ideation.
• Stressors related to finances, relationships, increased substance use, and COVID-19 exposure.

## 1 Introduction

Globally, almost 800,000 people die every year from suicide (World Health Organization, 2014). In Canada, approximately 4000 people die from suicide annually, while 2.5% of the population reports past-year suicidal thoughts (Public Health Agency of Canada [PHAC], 2016). Research further indicates that suicidal risk is not evenly distributed across the population, due to structural and social factors (e.g., age, gender, mental illness, those who live alone and are unmarried, LGBT2Q+, Indigenous, etc.) that increase risk via harmful and inequitable conditions that limit access to the determinants of good health for specific populations and groups (Findlay, 2017; Have et al., 2009; Nock et al., 2008). Since the onset of the COVID-19 pandemic, suicide risk has also been found to be elevated among those who have lost a job, are unemployed, or have a reduced household income (Findlay, 2017; Have et al., 2009; Kumar & Tjepkema, 2019; Teuton, 2018), and among those who experience interpersonal violence (Faust et al., 2021) or have increased their substance use (Canady, 2021; Faust et al., 2021).

While death by suicide has been referred to as a lagging indicator when assessing the severity of population mental health challenges, the prevalence of suicidal ideation (i.e., thoughts of suicide) is considered a more time-sensitive indicator for potential suicide risk (Botchway & Fazel, 2021; Nordt, Warnke, Seifritz, & Kawohl, 2015). In a global context, various risk factors have been identified for suicidal ideation, including age (<35 years-old) (Nock et al., 2008); presence of a mental health condition (Have et al., 2009; Nock et al., 2008; Teuton, 2018); living alone and/or being unmarried (Have et al., 2009; Nock et al., 2008); and LGBT2Q+ identity (Canady, 2021; Casey, 2019), to name a few. In Canada, research has further shown that being Indigenous (Kumar & Tjepkema, 2019) or a woman, is associated with an increased likelihood of experiencing suicidal thoughts (Ferro, Gorter, & Boyle, 2015).

During the first year of the COVID-19 pandemic, numerous studies documented a rising prevalence in suicidal thoughts among various population groups (Dubé, Smith, Sherry, Hewitt, & Stewart, 2021; Fitzpatrick, Harris, & Drawve, 2020; Jenkins et al., 2021; Xiong et al., 2020). This trend occurs alongside increases in depression and anxiety, which are associated with higher risk of suicidal ideation (Czeisler et al., 2020; Sher, 2020). According to Dubé and colleagues’ (2021) meta-analysis of 54 studies, an increase in suicidal ideation across all studies has been observed, with global pre-pandemic levels of ∼5% (Liu, Bettis, & Burke, 2020) increasing to 10.8% during the pandemic (Dubé et al., 2021). Furthermore, several mental health-related risk factors for suicidal ideation have increased in the context of the COVID-19 pandemic, including distress, insomnia, isolation, loneliness, diminished sense of control, financial loss/strain, work pressure, inability to return to work, hopelessness, and helplessness (Statistics Canada, 2020; Canady, 2021; Mental Health Commission of Canada, 2020; Sher, 2020), as well as “CORONEX” – a term identified by da Silva (2021) referring to coronavirus “presence” or exhaustion. Within Canada, there has been a surge in reported calls to Canadian distress centres, including the Canada Suicide Prevention Service (up 50% in 2020), as well as a high demand for virtual crisis support (Basky, 2021; Mental Health Commission of Statistics Canada, 2020). Crisis line volunteers have also reported an increase in intense interactions, with 62% more active rescues (responder response to emergency calls due to imminent risk of harm or suicide in-progress) (Wright, 2020 in Mental Health Commission of Statistics Canada, 2020). Whether or not suicidal ideation advances to suicidal behaviours, there has been considerable impact on the burden of population-level mental health.

At present, mental health challenges continue to rise, with concerns about the extent of suicidal ideation during and beyond the pandemic. Although it is not possible to know the total number of deaths by suicide directly related to the COVID-19 pandemic, people worldwide are experiencing and reporting increased suicidal ideation and mental health challenges (Czeisler et al., 2020; Fitzpatrick et al., 2020; López Steinmetz et al., 2020; Mental Health Commission of Statistics Canada, 2020; Tanaka & Okamoto, 2021). Moreover, those who experience financial-stressors (Canetto, 2015; Elbogen, Lanier, Blakey, Wagner, & Tsai, 2021; Kousoulis et al., 2020; Reger, Stanley, & Joiner, 2020) and/or relationship violence or stressors (Canetto, 2015; Iob, Steptoe, & Fancourt, 2020; Monteith, Holliday, Brown, Brenner, & Mohatt, 2020; Soole, Kõlves, & De Leo, 2015; Usher, Bhullar, Durkin, Gyamfi, & Jackson, 2020) have increased risk of suicidal ideation. Given the rise in suicidal ideation and other suicide-related risk factors, there is a pressing need for research monitoring the extent of suicidal ideation and associated correlates to guide targeted intervention and prevention efforts. Therefore, the aims of this paper are: (1) to quantify the extent of suicidal ideation due to the COVID-19 pandemic in the adult population in Canada and within key sub-groups, and (2) to identify sociodemographic and pandemic-related stressors that may be associated with increased risk of suicidal ideation.

## 2 Methods

### 2.1 Survey Development and approach

This study was developed in partnership between researchers at the University of British Columbia (UBC) and the Canadian Mental Health Association (CMHA), with international collaboration from the Mental Health Foundation in the United Kingdom. Team members represent a variety of disciplines, work-sectors, and lived experiences, benefiting the development of the survey, and creating pathways for impact. Survey items were first informed by the Mental Health Foundation's monitoring study, first commissioned in March 2020, which incorporated input from people with lived experience of mental health conditions and research on mental health impacts of past pandemics (Kousoulis et al., 2020). Modifications were then made by the UBC research team, in consultation with CMHA collaborators, to reflect the Canadian context.

Data are derived from three rounds of surveying conducted as part of the *Assessing the Impacts of COVID-19 on Mental Health* monitoring study examining the mental health impacts of the pandemic in Canada. Round 1 data were collected May 14–29, 2020, at a time when many Canadian provinces were opening back up following initial public health restrictions. Round 2 data were collected September 14–21, 2020, marking the end of summer months during which many public health restrictions were eased. At this time, many young people were returning to schools for the first time since spring. Round 3 data were collected January 22–28, 2021, following the winter holidays where many provinces had implemented strict public health restrictions, and vaccine rollout was in its early stages. National polling vendor Maru-Matchbox distributed surveys to their online panel consisting of approximately 125,000 members. Surveys, available in English and French (Canada's two official languages), were sent to a random selection of panel members, stratified by current Canadian census-informed socioeconomic characteristics (age, gender, household income, and province/territory). The sampling approach was further adjusted for response propensity to support representativeness according to the aforementioned census-informed socioeconomic stratifications.

Response-to-invitation ratios were 32% (Round 1), 36% (Round 2), and 36% (Round 3) resulting in 9061 completed surveys. Of these, 1993 (22%) were from respondents who participated in more than one survey round, resulting in a final sample of 7068 unique participants. A comparison of participant characteristics for those who did, versus those who did not, participate in multiple survey rounds indicated that the following groups were more likely to have repeat participation: <65 years of age, a household income greater than $100k, live in the province of Quebec, or experienced suicidal thoughts in the previous 2 weeks (See Supplementary Table 1 for details).

### 2.2 Ethics

Ethical approval for this study was obtained by the University of British Columbia Behavioural Research Ethics Board (H20-01273). Participants provided consent online prior to beginning the survey and were offered information on how to access mental health supports if needed. Maru/Matchbox provided a small honorarium for all participants who completed the survey.

### 2.3 Measures

#### 2.3.1 Suicidal ideation

Suicidal ideation was assessed by asking participants “Have you done or experienced any of the following, as a result of the COVID-19 pandemic in the past two weeks?”, including “experienced suicidal thoughts or feelings”. Response options were “Yes”, “No” and “Prefer not to say”.

#### 2.3.2 Sociodemographic factors

The following sociodemographic characteristics were collected: age; household income; province of residence; marital status; LGBT2Q+ identity; Indigenous origins; pre-existing (i.e., prior to COVID-19) mental health condition; and gender.

#### 2.3.3 Pandemic-related stressors

Pandemic-related stressors were divided into four categories: financial stressors, relationship stressors, increased substance use, and COVID-19 exposure stressors. Financial stressors and relationship stressors were derived from a question that asked whether participants had been stressed or worried about a variety of different stressful circumstances as a result of the COVID-19 pandemic in the past two weeks. Two financial stressors were assessed (1) “financial concerns (e.g., going into debt, ability to pay bills, long-term economic impacts, etc.):” and (2) “losing my job/loss of my job”. Two relationship stressors were assessed: (1) “experiencing relationship challenges with my partner” and (2) “being safe from physical or emotional domestic violence”. Response options for these items included “Yes”, “No”, “Don't know”, “Not applicable”, and “Prefer not to say”. Responses were dichotomized for analysis as “Yes” and “Not yes” which included “No”, “Don't know”, “Not applicable”, and “Prefer not to say”.

Increased substance use was assessed by asking participants how a series of substance use behaviors had been impacted by the COVID-19 pandemic. Five behaviors were assessed: (1) drinking alcohol, (2) use of tobacco products (e.g., cigarettes, cigars, chewing tobacco, vaping, etc.), (3) use of cannabis products, (4) use of prescribed medication, and (5) use of other psychoactive substances (e.g., cocaine, heroin). Response options included “More”, “Less”, “No change”, “Not applicable”, and “Prefer not to say”; responses were dichotomized for analysis as “More” and “Not more”, which included “Less”, “No change”, “Not applicable” and “Prefer not to say”.

COVID-19 exposure stressors were assessed by asking participants how they had been affected by the disease at any point during the pandemic. Four exposure options were assessed: (1) having tested positive for COVID-19, (2) having someone in their household test positive for COVID-19, (3) having a family member/loved one living at a different address test positive for COVID-19, and (4) fear of getting severely sick or dying in the past two weeks. Response options for (1) to (3) were “Yes” and “No”. Response options for (4) included “Yes”, “No”, “Don't know”, “Not applicable” and “Prefer not to say”. Responses were dichotomized for analysis as “Yes” and “Not yes” which included “No”, “Don't know”, “Not applicable”, and “Prefer not to say”.

### 2.4 Statistical analysis

Descriptive statistics were used to characterize the sample and quantify the extent of COVID-19 related suicidal ideation across the three survey rounds. Bivariate analyses (chi-square tests) were used to examine proportions of respondents reporting suicidal ideation by sociodemographic factors and pandemic-related stressors. To meet the assumptions of independent samples for chi square and subsequent regression analyses, we only included the earliest completed survey if participants had completed multiple survey rounds.

Multivariable logistic regression was used to examine the associations between COVID-19 related suicidal ideation and sociodemographic factors, as well as pandemic-related stressors within each of the four defined factor categories. To facilitate inclusion in the regression models, a “Non-cisgender” category was created containing respondents who identified as transgender woman, transgender man, or non-binary. Those who responded with Two-Spirit, not listed, and prefer not to answer were not included in the multivariate modeling, owing to important differences in the Two-Spirit (Indigenous) conceptualization of gender/sexuality, which differs from western identities (Pruden & Salway, 2020). Unadjusted models were used to examine the independent associations between each indicator variable and suicidal ideation. To account for potential confounding by sociodemographic differences, as well as changes in suicidal ideation over time, separate adjusted models were used to examine the independent associations between each indicator variable within each stressor category and suicidal ideation, adjusting for sociodemographic factors and survey round. Results are presented as odds ratios with 95% confidence intervals. All analyses were performed using SPSS version 27 (IBM Corp, 2020).

## 3 Results

Of the 7068 unique participants, 66 (1%) selected “Prefer not to say” for the assessment of suicidal ideation. These participants were excluded from subsequent analyses resulting in a final sample of 7002. Participants who selected “Prefer not to say” were more likely to be in the cisgender male or non-cisgender categories, aged 18–34 years, and have a household income less than $25k (see Supplementary Table 2 for details). In the final sample of 7002 participants, 6.2% of respondents (n = 433) indicated that within the past two weeks, they had experienced suicidal thoughts or feelings as a result of the COVID-19 pandemic. Table 1 characterizes the sample and presents the proportion of the sample experiencing COVID-19 related suicidal ideation by sociodemographic factors. Suicidal ideation in the previous two weeks was more frequently reported among those who were not cisgender (e.g., transgender women), were <65 years-old, single, identified as Indigenous or LGBT2Q+, or who had a pre-existing mental health condition.

**Table 1:** Sample characteristics, overall and by reporting having experienced suicidal thoughts or feelings in the previous 2 weeks (n, row %).

	Overall study sample	Experienced suicidal thoughts or feelings in the previous 2 weeks		
	(n, column %) (n = 7002 a)	No (n = 6569, 93.8%)	Yes (n = 433, 6.2%)	Chi-square test p-value
Gender				<.001
Cisgender man	3350 (47.8)	3150 (48.0)	200 (46.2)	
Cisgender woman	3577 (51.1)	3366 (51.2)	211 (48.7)	
Transgender woman	22 (0.3)	11 (0.2)	11 (2.5)	
Transgender man	7 (0.1)	7 (0.1)	0 (0.0)	
Non-binary	20 (0.3)	14 (0.2)	6 (1.4)	
Two-spirit	8 (0.1)	6 (0.1)	2 (0.5)	
Not listed	4 (0.1)	3 (0.0)	1 (0.2)	
Prefer not to answer	14 (0.2)	12 (0.2)	2 (0.5)	
Age				<.001
18–34 years	874 (12.5)	763 (11.6)	111 (25.6)	
35–64 years	3942 (56.3)	3668 (55.8)	274 (63.3)	
65+ years	2186 (31.2)	2138 (32.5)	48 (11.1)	
Household income				<.001
Under $25k	503 (7.3)	438 (6.8)	65 (15.1)	
$25k=<$50k	1258 (18.2)	1173 (18.1)	85 (19.7)	
$50k=<$100k	2456 (35.6)	2317 (35.8)	139 (32.3)	
$100k +	2688 (38.9)	2546 (39.3)	142 (32.9)	
Province				.062
Alberta	852 (12.2)	791 (12.0)	61 (14.1)	
British Columbia	1048 (15.0)	985 (15.0)	63 (14.5)	
Manitoba	262 (3.7)	248 (3.8)	14 (3.2)	
New Brunswick	147 (2.1)	143 (2.2)	4 (0.9)	
Newfoundland and Labrador	103 (1.5)	96 (1.5)	7 (1.6)	
Nova Scotia	360 (5.1)	336 (5.1)	24 (5.5)	
Ontario	2643 (37.7)	2464 (37.5)	179 (41.3)	
Prince Edward Island	27 (0.4)	26 (0.4)	1 (0.2)	
Quebec	1302 (18.6)	1245 (19.0)	57 (13.2)	
Saskatchewan	232 (3.3)	211 (3.2)	21 (4.8)	
Territories b	26 (0.4)	24 (0.4)	2 (0.5)	
Marital status				<.001
Single	1368 (19.5)	1216 (18.5)	152 (35.1)	
Married or partnered	4655 (66.5)	4433 (67.5)	222 (51.3)	
Separated, divorced, widowed	979 (14.0)	920 (14.0)	59 (13.6)	
LGBT2Q+ c				<.001
Yes or unsure	495 (7.1)	410 (6.3)	85 (20.0)	
No	6471 (92.9)	6130 (93.7)	341 (80.0)	
Ethnicity d				<.001
Indigenous	209 (3.1)	177 (2.8)	32 (7.9)	
Not Indigenous	6531 (96.9)	6156 (97.2)	375 (92.1)	
Pre-existing mental health condition e				<.001
Yes	1182 (17.0)	955 (14.6)	227 (54.3)	
No	5755 (83.0)	5564 (85.4)	191 (45.7)	
Study round				0.001
Round 1 (May 2020)	2968 (42.4)	2792 (42.5)	176 (40.6)	
Round 2 (September 2020)	2454 (35.0)	2271 (34.6)	183 (42.3)	
Round 3 (January 2021)	1580 (22.6)	1506 (22.9)	74 (17.1)	

a Final sample is composed of 7068 participants who participated in only one survey round; descriptive analyses exclude 66 participants who selected “Prefer not to say” for the assessment of suicidal ideation.

b Includes Northwest Territories, Nunavut, and Yukon.

c Participants were asked, “Do you identify as being LGBT2Q+ (lesbian, gay, bisexual, trans, two-spirit, queer, etc.)?”

d Participants were asked, “What is your family ethnicity” and were able to select multiple options. Respondents were classified as Indigenous if they selfidentified having a family of Indigenous origins, even if they identified additional ethnic categories.

e Participants were asked, “Do you identify as a person who has a pre-existing (prior to COVID-19) mental health condition?”

Table 2 presents the distribution of the measured pandemic-related stressors across the full sample and for those who identified experiencing suicidal ideation. Across the full sample, a large proportion of respondents reported financial stressors including stress or worry about financial concerns (34.5%), relationship stressors including worry about experiencing relationship challenges (17.1%), and increased alcohol use (17.1%). With respect to other substance use, <7% reported increased use of individual substances other than alcohol (i.e., tobacco, cannabis, prescribed medications, and other psychoactive substances). In terms of COVID-19 exposure stressors, a small proportion (<6%) of respondents reported that they or someone in their family or household had tested positive for COVID-19, however 36.9% reported experiencing a fear of getting severely sick or dying. Those who reported experiencing suicidal thoughts or feelings in the past two weeks were more likely to also report experiencing each of the stressors except for having a family member/loved one living at a different address testing positive for COVID-19.

**Table 2:** Pandemic-related stressors, overall and by reporting having experienced suicidal thoughts or feelings in the previous 2 weeks (n, column %).

Suicidal Ideation risk factor group	Indicators	Overall study sample	Experienced suicidal thoughts or feelings in the previous 2 weeks		
		(n, column %) (n = 7002 a)	No (n = 6569, 93.8%)	Yes (n = 433, 6.2%)	Chi-square test p-value
Financial stressors	Financial concerns				<.001
	Yes	2415 (34.5)	2139 (32.6)	276 (63.7)	
	Not yes	4587 (65.5)	4430 (67.4)	157 (36.3)	
	Losing my job				<.001
	Yes	1395 (19.9)	1209 (18.4)	186 (43.0)	
	Not yes	5607 (80.1)	5360 (81.6)	247 (57.0)	
Relationship stressors	Experiencing relationship challenges				<.001
	Yes	1200 (17.1)	1035 (15.8)	165 (38.1)	
	Not yes	5802 (82.9)	5534 (84.2)	268 (61.9)	
	Being safe from physical or emotional domestic violence				<.001
	Yes	565 (8.1)	491 (7.5)	74 (17.1)	
	Not yes	6437 (91.9)	6078 (92.5)	359 (82.9)	
Increased substance use	Drinking alcohol				<.001
	More	1198 (17.1)	1061 (16.2)	137 (31.6)	
	Not more	5804 (82.9)	5508 (83.8)	296 (68.4)	
	Use of tobacco products				<.001
	More	379 (5.4)	298 (4.5)	81 (18.7)	
	Not more	6623 (94.6)	6271 (95.5)	352 (81.3)	
	Use of cannabis products				<.001
	More	463 (6.6)	356 (5.4)	107 (24.7)	
	Not more	6539 (93.4)	6213 (94.6)	326 (75.3)	
	Use of prescribed medication				<.001
	More	278 (4.0)	193 (2.9)	85 (19.6)	
	Not more	6724 (96.0)	6376 (97.1)	348 (80.4)	
	Use of other psychoactive substances				<.001
	More	90 (1.3)	53 (0.8)	37 (8.5)	
	Not more	6912 (98.7)	6516 (99.2)	396 (91.5)	
COVID-19 stressors	Tested positive for COVID-19				<.001
	Yes	48 (0.7)	26 (0.4)	22 (5.1)	
	No	6954 (99.3)	6543 (99.6)	411 (94.9)	
	Someone in my household tested positive for COVID-19				<.001
	Yes	50 (0.7)	33 (0.5)	17 (3.9)	
	No	6952 (99.3)	6536 (99.5)	416 (96.1)	
	Family member/loved one living at a different address tested positive for COVID-19				.114
	Yes	300 (4.3)	275 (4.2)	25 (5.8)	
	No	6702 (95.7)	6294 (95.8)	408 (94.2)	
	Fear of getting severely sick or dying				<.001
	Yes	2583 (36.9)	2356 (35.9)	227 (52.4)	
	Not yes	4419 (63.1)	4213 (64.1)	206 (47.6)	

a Final sample is composed of 7068 participants who participated in only one survey round; descriptive analyses exclude 66 participants who selected “Prefer not to say” for the assessment of suicidal ideation.

Results of the logistic regression models that included sociodemographic factors and study round are presented in Table 3. Compared to cisgender women, cisgender men reported higher odds of experiencing suicidal thoughts or feelings in the past two weeks (Adjusted Odds Ratio (AOR) = 1.28, 95% Confidence Interval (CI): 1.02, 1.61), as did those in the non-cisgender category (AOR = 2.88, 95% CI: 1.16, 7.15). Being 18–34 years old (AOR = 3.23, 95% CI: 2.13, 4.89), single (AOR = 1.65, 95% CI: 1.26, 2.17), having Indigenous origins (AOR = 1.88, 95% CI: 1.21, 2.92), identifying as LGBT2Q+ (AOR = 1.67, 95% CI: 1.23, 2.28), and having a pre-existing mental health condition (AOR = 5.32, 95% CI: 4.23, 6.70) were all associated with increased odds of experiencing COVID-related suicidal thoughts or feelings in the past two weeks. There was also an association between suicidal ideation and study round, with the odds of reporting suicidal thoughts or feelings being higher in Round 2 compared to Round 1 (AOR = 1.54, 95% CI: 1.20, 1.96). The results of a set of sensitivity analyses for the same models, with respondents who answered the suicidal ideation question with “Prefer not to say” all coded as “Yes” and then coded as “No”, yielded the same pattern of results as the analyses with these respondents removed (see Supplementary Tables 3a and 3b respectively).

**Table 3:** Results of logistic regression models for reporting having experienced COVID-related suicidal thoughts or feelings in the previous 2 weeks – Sample characteristics (odds ratio, 95% confidence interval) (n = 6976 a).

	Unadjusted models	Adjusted model (including all sociodemographic factors and study round)
Gender		
Cisgender man	1.01 (0.83, 1.24)	1.28 (1.02, 1.61)
Cisgender woman	Reference	Reference
Non-cisgender b	8.47 (4.63, 15.51)	2.88 (1.16, 7.15)
Age		
18–34 years	6.41 (4.52, 9.09)	3.23 (2.13, 4.89)
35–64 years	3.27 (2.40, 4.47)	2.36 (1.68, 3.31)
65+ years	Reference	Reference
Household income		
Under $25k	2.66 (1.94, 3.63)	1.29 (0.87, 1.89)
$25k=<$50k	1.31 (0.99, 1.73)	1.05 (0.76, 1.46)
$50k=<$100k	1.06 (0.83, 1.35)	0.89 (0.68, 1.17)
$100k +	Reference	Reference
Province		
Alberta	1.06 (0.79, 1.43)	0.97 (0.69, 1.37)
British Columbia	0.88 (0.65, 1.18)	1.03 (0.74, 1.42)
Manitoba	0.78 (0.44, 1.36)	0.78 (0.42, 1.44)
New Brunswick	0.39 (0.14, 1.05)	0.50 (0.18, 1.39)
Newfoundland and Labrador	1.00 (0.46, 2.19)	1.58 (0.68, 3.64)
Nova Scotia	0.98 (0.63, 1.53)	0.81 (0.50, 1.33)
Ontario	Reference	Reference
Prince Edward Island	0.53 (0.07, 3.92)	0.51 (0.06, 4.07)
Quebec	0.63 (0.46, 0.86)	0.85 (0.61, 1.19)
Saskatchewan	1.37 (0.85, 2.20)	1.23 (0.71, 2.12)
Territories c	1.15 (0.27, 4.89)	1.46 (0.30, 7.02)
Marital status		
Single	2.55 (2.05, 3.16)	1.65 (1.26, 2.17)
Married or partnered	Reference	Reference
Separated, divorced, widowed	1.28 (0.95, 1.73)	1.33 (0.94, 1.88)
LGBT2Q+: Yes or unsure d	3.63 (2.80, 4.72)	1.67 (1.23, 2.28)
Ethnicity: Indigenous e	2.94 (1.98, 4.37)	1.88 (1.21, 2.92)
Pre-existing mental health condition: Yes f	6.89 (5.61, 8.45)	5.32 (4.23, 6.70)
Study round		
Round 1 (May 2020)	Reference	Reference
Round 2 (September 2020)	1.27 (1.02, 1.57)	1.54 (1.20, 1.96)
Round 3 (January 2021)	0.78 (0.59, 1.03)	1.02 (0.75, 1.40)

a Final sample is composed of 7068 participants who participated in only one survey round; regression analyses exclude 66 participants who selected “Prefer not to say” in the assessment of suicidal ideation and 26 participants who selected “Two-Spirit”, “Not listed”, or “Prefer not to answer” in the gender question.

b Includes transgender woman, transgender man, and non-binary.

c Includes Northwest Territories, Nunavut, and Yukon.

d Participants were asked, “Do you identify as being LGBT2Q+ (lesbian, gay, bisexual, trans, two-spirit, queer, etc.)?”

e Participants were asked, “What is your family ethnicity” and were able to select multiple options. Respondents were classified as Indigenous if they selfidentified having a family of Indigenous origins, even if they identified additional ethnic categories.

f Participants were asked, “Do you identify as a person who has a pre-existing (prior to COVID-19) mental health condition?”

Results of the logistic regression models for each stressor and substance use variable with adjustment for sociodemographic characteristics are presented in Table 4. All indicators in the financial and relationship stressor categories were associated with higher odds of suicidal ideation in the unadjusted and adjusted models. For example, odds of suicidal ideation were higher for those who reported stress or worry about financial concerns (AOR = 2.48, 95% CI: 1.97, 3.13), job loss (AOR = 2.61, 95% CI: 2.07, 3.29), relationship challenges (AOR = 2.88, 95% CI: 2.26, 3.67), and being safe from physical or emotional domestic violence (AOR = 2.03, 95% CI: 1.47, 2.80).

**Table 4:** Results of logistic regression models for reporting having experienced COVID-related suicidal thoughts or feelings in the previous 2 weeks – Pandemic-related stressors (odds ratio, 95% confidence interval) (n = 6976 a).

	Unadjusted models	Adjusted models (individual models for each stressor that include adjustments for sociodemographic factors and study round)
Financial stressors		
Financial concerns	3.65 (2.97, 4.47)	2.48 (1.97, 3.13)
Losing their job	3.37 (2.76, 4.12)	2.61 (2.07, 3.29)
Relationship stressors		
Experiencing relationship challenges	3.32 (2.70, 4.07)	2.88 (2.26, 3.67)
Being safe from physical or emotional domestic violence	2.55 (1.95, 3.33)	2.03 (1.47, 2.80)
Increased substance use		
Drinking alcohol	2.39 (1.93, 2.96)	1.99 (1.56, 2.55)
Use of tobacco products	4.78 (3.65, 6.27)	2.56 (1.83, 3.57)
Use of cannabis products	5.60 (4.38, 7.16)	2.91 (2.18, 3.89)
Use of prescribed medication	8.04 (6.09, 10.62)	3.51 (2.48, 4.96)
Use of other psychoactive substances	11.25 (7.28, 17.39)	4.55 (2.57, 8.06)
COVID-19 stressors		
Tested positive for COVID-19	12.94 (7.22, 23.20)	4.85 (2.05, 11.45)
Someone in their household tested positive for COVID-19	7.67 (4.19, 14.04)	1.88 (0.69, 5.14)
Family member/loved one living at a different address tested positive for COVID-19	1.42 (0.93, 2.16)	1.14 (0.68, 1.89)
Fear of getting severely sick or dying	1.98 (1.63, 2.41)	1.67 (1.34, 2.08)

b Includes transgender woman, transgender man, and non-binary.

a Final sample is composed of 7068 participants who participated in only one survey round; regression analyses exclude 66 participants who selected “Prefer not to say” in the assessment of suicidal ideation and 26 participants who selected “Two-Spirit”, “Not listed”, or “Prefer not to answer” in the gender question.

Increased substance use was also associated with higher odds of suicidal ideation. Specifically, odds of suicidal ideation were higher for those who reported drinking more alcohol (AOR = 1.99, 95% CI: 1.56, 2.55), or using more tobacco products (AOR = 2.56, 95% CI: 1.83, 3.57), cannabis products (AOR = 2.91, 95% CI: 2.18, 3.89), prescribed medication (AOR = 3.51, 95% CI: 2.48, 4.96), or psychoactive substances (AOR = 4.55, 95% CI: 2.57, 8.06).

Indicators in the COVID-19 exposure stressors category that were associated with higher odds of suicidal ideation included having previously tested positive for COVID-19 (AOR = 4.85, 95% CI: 2.05, 11.45) and reporting a fear of getting severely sick or dying (AOR = 1.67, 95% CI: 1.34, 2.08). The results of a set of sensitivity analyses for the same models with respondents who answered the suicidal ideation question with “Prefer not to say” all coded as “Yes” and then coded as “No” yielded the same pattern of results as the analyses with these respondents removed (see Supplementary Tables 4a and 4b respectively).

## 4 Discussion

Current research has generated limited knowledge on COVID-19 related suicidal ideation and the associated correlates. This investigation contributes to addressing this gap using data from a nationally representative Canadian sample across three time points during the first year of the pandemic. Exploring known risk factors for suicidal ideation and novel stressors related to the COVID-19 pandemic alongside sociodemographic correlates provides important data on *who* is most impacted. These findings are critical to informing responsive policy and practice solutions that address the key drivers of suicidality and suicide risk for the populations most impacted. Results indicate that certain sociodemographic characteristics were associated with higher odds of suicidal ideation. Additionally, stressors related to finances, relationships, and COVID-19 exposure, as well as increased substance use, also contributed to a greater likelihood of suicidal ideation, after adjusting for sociodemographic factors and survey round.

Our results identify key sociodemographic characteristics associated with higher odds of COVID-19 related suicidal ideation (Czeisler et al., 2020; Czeisler et al., 2021; Dubé et al., 2021; Fitzpatrick et al., 2020; Jenkins et al., 2021; López Steinmetz et al., 2020). These include being younger (18–34 years), living alone or being unmarried, experiencing a pre-existing mental health condition, and identifying as LGBT2Q+ or Indigenous. These results align with studies conducted prior to the pandemic, which have also identified increased suicidal ideation among those who belong to one or more of these groups/categories (Casey, 2019; Have et al., 2009; Kumar & Tjepkema, 2019; Nock et al., 2008; Teuton, 2018). Indeed, research on the social and structural determinants of mental health has long identified sociodemographic “risk” factors for mental ill health and suicidality. At a population level, these influences are theorized to operate through conditions of cumulative stress (Alegría, NeMoyer, Bagué, Wang, & Alvarez, 2018), which tend to cluster within groups or communities and create barriers to determinants of good health. Moreover, these sociodemographic characteristics tend to intersect with other identity factors and sociopolitical forces that impact experiences of suicidality (Blosnich, 2017; Canetto, 2015). At an individual level, this cumulative stress is further implicated in producing physiological and neurochemical changes in brain. These changes are associated with symptoms of depression and anxiety, which can increase risk for feelings of suicidality (Walker, Campbell, Dawson, & Egede, 2019).

Our findings related to the sociodemographic correlates of suicidality are further aligned with research conducted during the pandemic. For example, those who are younger (18–34 years) have been identified in other studies as one of the groups most likely to experience suicidal ideation (Czeisler et al., 2020; Czeisler et al., 2021; Dubé et al., 2021; Fitzpatrick et al., 2020; Jenkins et al., 2021; López Steinmetz et al., 2020). Also aligned was our finding that those who are living alone or who are not partnered tend to be more likely to experience suicidal thoughts (Fitzpatrick et al., 2020). Differences in suicidal thoughts and behaviours by race and/or ethnicity have also been documented. For example, those who identify as Indigenous have been described as two to four times more likely to report suicidal thoughts compared to their non-Indigenous counterparts (Fitzpatrick et al., 2020; Mental Health Commission of Statistics Canada, 2020). This is likely a result of the continued legacy of colonization that creates conditions (e.g., poverty, food insecurity, violence/abuse, disrupted social supports, etc.) in which suicide may feel like the better option for some (Walker et al., 2019). Moreover, while declines in overall suicide death rates have been identified during the early months of the pandemic, a recent study in the U.S. concluded that while the trend was encouraging, it “was due entirely to a significant 2.2% decrease in suicide among white people. No other racial or ethnic group saw a statistically significant change in suicide rates” (Stone, 2021 in; Kuehn, 2021, p. 1386). Indeed, multiple U.S. national surveys have shown higher rates of both suicide deaths and ideation among Indigenous populations (Fitzpatrick et al., 2020; Mitchell & Li, 2021; Polanco‐Roman & Miranda, 2021). Overall, our study highlights the importance of capturing disaggregated data, leading to meaningful understandings regarding current trends for those most inequitably impacted by suicidality due to the pandemic.

Our findings have also illuminated gender differences in experiences of suicidality during the pandemic. Specifically, our data indicate that cisgender men are more likely to report COVID-19 related suicidal ideation than cisgender women. This finding was surprising given that prior to the pandemic, women have consistently been identified as more likely to experience suicidal ideation compared to men (Ferro et al., 2015). However, these findings mirror those recently observed in the UK (Iob et al., 2020; O'Connor et al., 2021; Sueki & Ueda, 2021) and in the US (Czeisler et al., 2020; Elbogen et al., 2021). The impacts of the “shecession”, where job and income loss has been more heavily concentrated among women (Holpuch, 2020), as well as the “pulling together” phenomenon, associated with strengthened social connectedness and a unifying of communities, may be helpful areas for further inquiry to better understand this phenomenon (Ayers et al., 2021; Barr, Taylor-Robinson, Scott-Samuel, McKee, & Stuckler, 2012; Holpuch, 2020; Reeves, McKee, & Stuckler, 2014; Reger et al., 2020). Not unexpectedly, those who identified as non-cisgender were more likely than cisgender men and cisgender women to report suicidal ideation. This is consistent with findings from a global cross-sectional study (Jarrett et al., 2020) and US based online survey (Radusky et al., 2021), both of which have also identified non-cisgender groups as being among those at greatest risk for suicidal ideation during the pandemic. Given these findings, future research should continue to monitor the gendered patterns of suicidal ideation. Moreover, research is critically needed to further capture trends in suicidal ideation among non-cisgender populations that were aggregated or excluded in our analyses (e.g., transgender, non-binary, Two-Spirit), as there are likely important differences in the impacts of the COVID-19 pandemic across these populations.

Beyond the sociodemographic characteristics associated with increased odds of COVID-19 related suicidal ideation, we also examined the impacts of various stressors. Like others, we found that financial stressors play an important role in suicidal thinking. For example, 13.3% of those who lost a job and 11.4% of those who reported financial concerns also indicated experiencing suicidal thoughts or feelings in the two weeks prior to participating in the survey – these stressors were also associated with increased odds of experiencing suicidal thoughts in the adjusted regression models. This is not surprising, as research has long demonstrated the strong relationship between financial or economic concerns and experiences of suicidal thinking and action (McIntyre & Lee, 2020; Reeves et al., 2014; Stuckler, Basu, Suhrcke, Coutts, & McKee, 2009). Concerningly, other research further demonstrates that people from marginalized and/or underfunded communities, or who are unemployed, under economic stress, or dealing with food insecurity are disproportionately affected by suicide and suicidality (Carr et al., 2021; Elbogen et al., 2021; Fitzpatrick et al., 2020; Kousoulis et al., 2020; McAuliffe et al., 2021; Reger et al., 2020). Additionally, our data demonstrate having a pre-existing mental health condition as a key risk factor for suicidal thinking. Indeed, even after adjusting for sociodemographic characteristics, those who had a pre-existing mental health condition had much higher odds of suicidal ideation compared to those without. While much of our data aligns with previous COVID-19 studies, our study differed in the breadth of correlates for suicidal thinking examined as well as the disaggregation of data to identify impacts at the level of population sub-groups.

In terms of relationship stressors, both experiencing relationship challenges and reporting worry about being safe from physical or emotional domestic violence were associated with increase odds of experiencing suicidal thoughts or feelings in the previous two weeks. Past research has identified that those with fewer social supports or who lack connectedness have increased risk of suicidal ideation (Elbogen et al., 2021). Strategies to support such groups are critical as they are often overlooked or “hidden” from view (Botchway & Fazel, 2021; Stone, 2021). Additionally, and not unexpectedly, our findings demonstrated a relationship between substance use and suicidality, with those who increased their substance use during the pandemic being more likely to report suicidal thinking compared to those who did not report this change in use. Such a relationship is well established in both the pandemic and pre-pandemic literature (Botchway & Fazel, 2021; Dubé et al., 2021; Fazel & Runeson, 2020; Yoshimasu, Kiyohara, Miyashita, & Hygiene, 2008).

To further understand independent effects of the pandemic, in our final model, we controlled for sociodemographic characteristics, including pre-existing mental health condition(s), and included variables related specifically to the COVID-19 pandemic. We found that respondents who tested positive for COVID-19 had nearly five times the odds of suicidal thoughts or feelings as compared to those who had not previously tested positive. Elsewhere we have reported on the elevated odds of suicidal ideation among those self-quarantining due to possible exposure (Daly et al., 2020). Studies have also shown that COVID-19 patients with post-COVID or “long haul” syndrome (i.e., symptoms extending beyond three weeks following first onset) may experience long-lasting psychological and neurobiological impacts. These reportedly include depression, anxiety, posttraumatic symptoms and PTSD, insomnia, and inflammatory brain damage; all of which are associated with increased suicide risk (Sher, 2020). Future research is needed to better understand the role of COVID-19 exposure and infection status on experiences of suicidal thinking.

### 4.1 Implications

Suicidal ideation is a concerning form of distress and those who are impacted are often hidden and go unrecognized (Jobes & Joiner, 2019). While suicidal ideation in Canada remains relatively low from that compared to a global perspective (Dubé et al., 2021), one hypothesis suggests this to be a result of the restrictions and limitations placed on accessibility to firearms (Leenaars, Moksony, Lester, & Wenckstern, 2003), as it is theorized that the ability to access lethal means increases the likelihood of shifting ideation to action (Klonsky, May, & Saffer, 2016). However, this investigation demonstrates that the proportion of Canadians experiencing suicidal thoughts or feelings has increased considerably during the COVID-19 pandemic, especially among groups impacted by issues of equity. Indeed, it was estimated in 2016 that 2.5% of the Canadian population experienced suicidal thoughts or feelings during the previous year (Public Health Agency of Canada [PHAC], 2016). In the present study, this proportion grew substantially, with 6.2% of survey respondents (averaged across rounds) identifying suicidal thoughts or feelings within the previous two weeks. Further, our analysis shows that the prevalence of COVID-19 related suicidal ideation is even higher for marginalized subpopulations.

As countries move toward pandemic recovery, understanding the increased prevalence of COVID-19 related suicidal ideation is crucial to planning appropriate long-term resources, supports, and policies to equitably support their populations’ well-being, including those who have been affected by post-COVID syndrome. Current responses being implemented in different nations include expanding the use of tele-mental health services (Czeisler et al., 2020; Gilmour, 2020), creating alternative treatment settings (private outdoor spaces, distribution of phones for access to tele-mental health services), addressing financial barriers through income and other government supports, and campaigns to raise awareness about available resources and how to access them (Mental Health Commission of Statistics Canada, 2020). Additionally, some locales have implemented a national suicide prevention number, such as 9-8-8 in Canada, though it will not be fully functioning until March 2023 (Basky, 2021). In the meantime, and for the long-term, there is a “need for socio-politically informed interventions at both the individual and population levels” that recognize “that suicide is sometimes (if not often) a response to policies, systems, and structures that produce vulnerabilities in the form of intergenerational trauma, racism, gender violence, toxic masculinities, social marginalization, and inequities” (White & Morris, 2019, p. 11). Thus, tailored and safe mental health care is critically needed for groups – such as those who are non-cisgender, LGBT2Q+, Indigenous, youth, or live with a mental health condition – who face these harmful conditions (Ferlatte et al., 2020; Wexler & Gone, 2012). Additionally, services that are responsive to the emerging needs of those who are living with post-COVID syndrome should be prioritized to help mitigate the effects on experiences of suicidality and to support these individuals in their recovery.

While there is an immediate need for increased access to mental health care, there is a further collective responsibility toward addressing the underlying structural contributors to suicidal ideation. This includes responding to issues of financial inequity, including through “emergency health care, mental health care, wage subsidies, supplemental income, and work retraining” (Barr et al., 2012; Reeves et al., 2014 in Mental Health Commission of Statistics Canada, 2020, p. 5). Moreover, social welfare policy interventions (e.g., government income support subsidies) should be considered alongside financial investments in employment opportunities and expanded mental health and substance use services. Building awareness in primary and community care contexts about risk factors for suicidal ideation, especially for populations living with health and social inequities will be important for mitigating the impacts through appropriate screening and bridging to meaningful supports (Mental Health Commission of Statistics Canada, 2020). Ongoing monitoring and evaluation, including longitudinal studies that track change in suicidal ideation over time, within individuals, is necessary to best support the inequitable mental health impacts of the COVID-19 pandemic.

### 4.2 Strengths and limitations

Sampling across sociodemographic stratifications with adjustment for response propensity was used to obtain a sample that is representative of the adult population in Canada by age, gender, province, and income (Statistics Canada, 2020). However, other population characteristics may not be representative, which may limit the generalizability of results to particular population sub-groups. For example, those experiencing technological barriers, including access to the internet, are not reflected in the sample. Furthermore, the survey was only conducted in Canada's two official languages, English and French, potentially missing those who are not proficient in reading these languages. Sampling methods including oversampling and community partner engagement were designed to maximize representativeness, but it is possible that not all populations were reached including individuals who are unhoused or vulnerably housed, and those with limited internet access. Another notable limitation is the cross-sectional study design, which does not support causal conclusions on the directionality of associations observed.

Additionally, while the purpose of this study was to examine associations between COVID-19 related suicidal ideation within the past two weeks and sociodemographic characteristics and stressors, we did not measure or control for pre-existing or ongoing suicidal ideation. Due to the sensitive nature of the topic, some participants may have been uncomfortable reporting certain experiences, such as suicidal thoughts or increased substance use. This may have affected our prevalence estimates. Moreover, as we were interested in capturing self-reported mental health experiences, we did not include clinical screening tools. Future research would benefit from the addition of standardized measures to identify clinically significant suicidality. We also excluded 66 participants (1.0%) from the analysis who indicated “Prefer not to say” to the suicidal ideation item. As noted in the Results (and documented in the associated Supplementary Table 2), we found that compared to those who answered this question, participants who selected “Prefer not to say” were more likely to be in the cisgender male or non-cisgender categories and to have a household income less than $25k. Although this finding does suggest that excluding participants who reply, “Prefer not to say” may bias our results, the results of sensitivity analyses with respondents who answered “Prefer not to say” all coded as “Yes” and then coded as “No” yielded the same pattern of results as the analyses with these respondents removed (see Supplementary Tables 3a, 3b, 4a and 4b). With the enduring nature and potential worsening of mental health impacts of the COVID-19 pandemic, ongoing monitoring is needed to continue to inform resource allocation and responsive service delivery while moving forward.

## 5 Conclusion

While research has identified stagnation or decline in suicide related deaths over the course of the pandemic, our research indicates that COVID-19 related suicidal ideation has significantly increased at the population-level in Canada, with higher prevalence noted for those within specific subpopulations – including those who are younger, single, are Indigenous, LGBT2Q+, not cisgender, or who have a pre-existing mental health condition. Likewise, a large proportion of respondents reported stressors related to finances, relationships, COVID-19 exposure, and increased substance use, which were associated with suicidal thoughts. Appropriate planning, resources, and policies are needed to protect and promote well-being and to mitigate suicidality through the pandemic recovery.

## Author's contributions

EJ and CM led the conceptualization of the study, including study design and data collection. CM, JP, CR, and KT led the analysis and interpretation of this manuscript. CM wrote the first draft, while JP, CR, KT, AS, TS, and EJ further contributed to the data interpretation and subsequent drafts this manuscript.

## Funding

The 10.13039/501100003250Canadian Mental Health Association (10.13039/501100003250CMHA) funded survey data collection through national polling vendor Maru/Matchbox.

## Ethics approval

Ethical approval for this study was obtained from the University of British Columbia Behavioural Research Ethics Board (H20-01273).

## Consent to participate

In advance of commencing the survey, participants provided consent online. Participants were provided with information about accessing mental health supports and received a small honorarium from Maru/Matchbox.

## Declaration of competing interest

CR reports receiving personal fees from the University of British Columbia during the conduct of this study. All other authors declare that they have no conflict of interest.

## References

[bib1] Alegría M., NeMoyer A., Bagué I.F., Wang Y., Alvarez K. (2018).

[bib2] Ayers J.W., Poliak A., Johnson D.C., Leas E.C., Dredze M., Caputi T. (2021). Suicide-related internet searches during the early stages of the COVID-19 pandemic in the US. JAMA network open.

[bib3] Barr B., Taylor-Robinson D., Scott-Samuel A., McKee M., Stuckler D. (2012). Suicides associated with the 2008-10 economic recession in England: Time trend analysis. BMJ.

[bib4] Basky G. (2021). Canada will have three-digit suicide prevention hotline by 2023.

[bib7] Blosnich J.R. (2017). Adult transgender care.

[bib8] Botchway S., Fazel S. (2021). Remaining vigilant about COVID-19 and suicide. The Lancet Psychiatry.

[bib9] Canady V.A. (2021). New literature review examines suicide risk during COVID‐19. Mental Health Weekly.

[bib10] Canetto S.S. (2015). Advancing the science of suicidal behavior.

[bib11] Carr M.J., Steeg S., Webb R.T., Kapur N., Chew-Graham C.A., Abel K.M., Ashcroft D.M. (2021). Effects of the COVID-19 pandemic on primary care-recorded mental illness and self-harm episodes in the UK: A population-based cohort study. The Lancet Public Health.

[bib12] Casey B. (2019).

[bib13] Czeisler M.É., Lane R.I., Petrosky E., Wiley J.F., Christensen A., Njai R., Barger L.K. (2020). Mental health, substance use, and suicidal ideation during the COVID-19 pandemic—United States, June 24–30, 2020. Morbidity and Mortality Weekly Report.

[bib14] Czeisler M.É., Wiley J.F., Facer-Childs E.R., Robbins R., Weaver M.D., Barger L.K., Rajaratnam S.M. (2021). Mental health, substance use, and suicidal ideation during a prolonged COVID-19–related lockdown in a region with low SARS-CoV-2 prevalence. Journal of Psychiatric Research.

[bib62] Daly Z., Slemon A., Chris G., Travis S., McAuliffe C., Gadermann A., Jenkins E. (2020). Associations between periods of COVID-19 quarantine and mental health in Canada. Psychiatry Research.

[bib15] Dubé J.P., Smith M.M., Sherry S.B., Hewitt P.L., Stewart S.H. (2021). Suicide behaviors during the COVID-19 pandemic: A meta-analysis of 54 studies. Psychiatry Research.

[bib16] Elbogen E.B., Lanier M., Blakey S.M., Wagner H.R., Tsai J. (2021).

[bib17] Faust J.S., Shah S.B., Du C., Li S.-X., Lin Z., Krumholz H.M. (2021). Suicide deaths during the CoViD-19 stay-at-home advisory in Massachusetts, March to may 2020. JAMA network open.

[bib18] Fazel S., Runeson B. (2020). Suicide. New England Journal of Medicine.

[bib19] Ferlatte O., Salway T., Oliffe J.L., Saewyc E.M., Holmes C., Schick L., Ho D. (2020). It is time to mobilize suicide prevention for sexual and gender minorities in Canada. Canadian Journal of Public Health.

[bib20] Ferro M.A., Gorter J.W., Boyle M.H. (2015). Trajectories of depressive symptoms in Canadian emerging adults. American Journal of Public Health.

[bib21] Findlay L. (2017). Statistics Canada.

[bib22] Fitzpatrick K.M., Harris C., Drawve G. (2020). How bad is it? Suicidality in the middle of the COVID‐19 pandemic. Suicide and Life-Threatening Behavior.

[bib23] Gilmour H. (2020). https://www150.statcan.gc.ca/n1/pub/45-28-0001/2020001/article/00011-eng.pdf.

[bib24] Have M.t., De Graaf R., Van Dorsselaer S., Verdurmen J., Van't Land H., Vollebergh W. (2009). Incidence and course of suicidal ideation and suicide attempts in the general population. Canadian Journal of Psychiatry.

[bib25] Holpuch A. (2020). https://www.theguardian.com/business/2020/aug/04/shecession-coronavirus-pandemic-economic-fallout-women?fbclid=IwAR1r1dMFHpjtFViHvobddS3d2nksQhhcWRAxek35nXvDt2PzBhFL1aweaio.

[bib26] IBM Corp (2020).

[bib27] Iob E., Steptoe A., Fancourt D. (2020). Abuse, self-harm and suicidal ideation in the UK during the COVID-19 pandemic. The British Journal of Psychiatry.

[bib28] Jarrett B.A., Peitzmeier S.M., Restar A., Adamson T., Howell S., Baral S. (2020).

[bib60] Jenkins E.K., McAuliffe C., Hirani S., Richardson C., Thomson K.C., McGuinness L., Gadermann A. (2021). A portrait of the early and differential mental health impacts of the COVID-19 pandemic in Canada: findings from the first wave of a nationally representative cross-sectional survey. Preventive Medicine.

[bib29] Jobes D.A., Joiner T.E. (2019). Reflections on suicidal ideation. Crisis.

[bib30] Klonsky E.D., May A.M., Saffer B.Y. (2016). Suicide, suicide attempts, and suicidal ideation. Annual Review of Clinical Psychology.

[bib31] Kousoulis A., McDaid S., Crepaz-Keay D., Solomon S., Lombardo C., Yap J., Davidson G. (2020). https://www.mentalhealth.org.uk/sites/default/files/MHF-covid-19-inequality-mental-health-briefing.pdf.

[bib32] Kuehn B.M. (2021). Disparities in suicide persist despite overall dip in US rate. Journal of the American Medical Association.

[bib33] Kumar M.B., Tjepkema M. (2019). Statistics Canada: Consumer policy research database.

[bib34] Leenaars A.A., Moksony F., Lester D., Wenckstern S. (2003). The impact of gun control (Bill C-51) on suicide in Canada. Death Studies.

[bib35] Liu R.T., Bettis A.H., Burke T.A. (2020). Characterizing the phenomenology of passive suicidal ideation: A systematic review and meta-analysis of its prevalence, psychiatric comorbidity, correlates, and comparisons with active suicidal ideation. Psychological Medicine.

[bib36] López Steinmetz L.C., Dutto Florio M.A., Leyes C.A., Fong S.B., Rigalli A., Godoy J.C. (2020). Levels and predictors of depression, anxiety, and suicidal risk during COVID-19 pandemic in Argentina: The impacts of quarantine extensions on mental health state. Psychology Health & Medicine.

[bib61] McAuliffe C., Daly Z., Black J., Gadermann A., Slemon A., Thomson K., Jenkins E. (2021). Food worry and mental health deterioration during the early months of the COVID-pandemic in Canada: Findings from a cross-sectional mental health monitoring survey. Canadian Journal of Public Health.

[bib37] Mental Health Commission of Canada (2020).

[bib38] Mitchell T.O., Li L. (2021). State-level data on suicide mortality during COVID-19 quarantine: Early evidence of a disproportionate impact on racial minorities. Psychiatry Research.

[bib39] Nock M.K., Borges G., Bromet E.J., Alonso J., Angermeyer M., Beautrais A., Gluzman S. (2008). Cross-national prevalence and risk factors for suicidal ideation, plans and attempts. The British Journal of Psychiatry.

[bib40] Nordt C., Warnke I., Seifritz E., Kawohl W. (2015). Modelling suicide and unemployment: A longitudinal analysis covering 63 countries, 2000–11. The Lancet Psychiatry.

[bib41] O'Connor R.C., Wetherall K., Cleare S., McClelland H., Melson A.J., Niedzwiedz C.L., Scowcroft E. (2021). Mental health and well-being during the COVID-19 pandemic: Longitudinal analyses of adults in the UK COVID-19 mental health & wellbeing study. The British Journal of Psychiatry.

[bib42] Polanco‐Roman L., Miranda R. (2021). A cycle of exclusion that impedes suicide research among racial and ethnic minority youth. Suicide and Life-Threatening Behavior.

[bib64] Pruden H., Salway T. (2020). https://cihr-irsc.gc.ca/e/52214.html.

[bib65] Public Health Agency of Canada [PHAC] (2016). https://www.canada.ca/en/public-health/services/publications/healthy-living/suicide-canada-key-statistics-infographic.html.

[bib43] Radusky P.D., Cardozo N., Duarte M., Fabian S., Frontini E., Sued O. (2021). Mental health, substance use, experiences of violence, and access to health care among transgender and non-binary people during the COVID-19 lockdown in Argentina. International Journal of Transgender Health.

[bib44] Reeves A., McKee M., Stuckler D. (2014). Economic suicides in the great recession in Europe and North America. The British Journal of Psychiatry.

[bib45] Reger M.A., Stanley I.H., Joiner T.E. (2020). Suicide mortality and coronavirus disease 2019—a perfect storm?. JAMA psychiatry.

[bib46] Sher L. (2020). The impact of the COVID-19 pandemic on suicide rates. QJM: International Journal of Medicine.

[bib47] da Silva J.A.T. (2021). Corona exhaustion (CORONEX): COVID-19-induced exhaustion grinding down humanity. Current Research in Behavioral Sciences.

[bib48] Statistics Canada (2020). https://www12.statcan.gc.ca/census-recensement/2016/dp-pd/index-eng.cfm.

[bib49] Stone D.M. (2021).

[bib50] Stuckler D., Basu S., Suhrcke M., Coutts A., McKee M. (2009). The public health effect of economic crises and alternative policy responses in Europe: An empirical analysis. The Lancet.

[bib51] Sueki H., Ueda M. (2021).

[bib52] Teuton J. (2018). http://www.healthscotland.scot/media/1712/social-isolation-and-loneliness-in-scotland-a-review-of-prevalence-and-trends.pdf.

[bib53] Walker R.J., Campbell J.A., Dawson A.Z., Egede L.E. (2019). Prevalence of psychological distress, depression and suicidal ideation in an indigenous population in Panamá. Social Psychiatry and Psychiatric Epidemiology.

[bib54] Wexler L.M., Gone J.P. (2012). Culturally responsive suicide prevention in indigenous communities: Unexamined assumptions and new possibilities. American Journal of Public Health.

[bib55] White J., Morris J. (2019). Re-thinking ethics and politics in suicide prevention: Bringing narrative ideas into dialogue with critical suicide studies. International Journal of Environmental Research and Public Health.

[bib56] World Health Organization (2014). https://www.who.int/publications/i/item/preventing-suicide-a-global-imperative.

[bib57] Wright T. (2020). https://www.thestar.com/news/canada/2020/04/27/crisis-lines-in-canada-face-volunteer-cash-crunch-even-as-covid-19-drives-surge-in-calls.html.

[bib58] Xiong J., Lipsitz O., Nasri F., Lui L.M.W., Gill H., Phan L., McIntyre R.S. (2020). Impact of COVID-19 pandemic on mental health in the general population: A systematic review. Journal of Affective Disorders.

[bib59] Yoshimasu K., Kiyohara C., Miyashita K. (2008). Suicidal risk factors and completed suicide: meta-analyses based on psychological autopsy studies. Environmental Health and Preventive Medicine.

