# Supplementary material for: Correlates of suicidal ideation related to the COVID-19 Pandemic: Repeated cross-sectional nationally representative Canadian data
Source: SSM Popul Health. 2021 Dec 9;16:100988. doi: 10.1016/j.ssmph.2021.100988 (PMC8656176; doi:10.1016/j.ssmph.2021.100988)
Supplement: Multimedia component 1 [file mmc1.pdf]

Supplementary Table 1: Comparison of participants who did and did not complete multiple survey rounds (n, row %)

	**Overall study sample**	**Completed multiple survey rounds**		
	**(n, column %)** (n=9061)	No (n=7068, 78.0%)	Yes (n=1993, 22.0%)	Chi-square test p-value
Gender				.049
Cisgender man	4313 (47.8)	3385 (48.1)	928 (46.8)	
Cisgender woman	4637 (51.4)	3605 (51.2)	1032 (52.0)	
Non-cisgender [a]	76 (0.8)	51 (0.7)	25 (1.3)	
Age				<.001
18-34 years	1240 (13.7)	888 (12.6)	352 (17.7)	
35-64 years	5296 (58.4)	3981 (56.3)	1315 (66.0)	
65+ years	2525 (27.9)	2199 (31.1)	326 (16.4)	
Household income				<.001
Under $25k	659 (7.4)	514 (7.4)	145 (7.4)	
$25k=<$50k	1585 (17.8)	1270 (18.2)	315 (16.1)	
$50k=<$100k	3101 (34.8)	2475 (35.5)	626 (32.0)	
$100k +	3578 (40.1)	2709 (38.9)	869 (44.5)	
Province				<.001
Alberta	1066 (11.8)	861 (12.2)	205 (10.3)	
British Columbia	1309 (14.4)	1055 (14.9)	254 (12.7)	
Manitoba	320 (3.5)	266 (3.8)	54 (2.7)	
New Brunswick	194 (2.1)	149 (2.1)	45 (2.3)	
Newfoundland and Labrador	136 (1.5)	103 (1.5)	33 (1.7)	
Nova Scotia	435 (4.8)	365 (5.2)	70 (3.5)	
Ontario	3419 (37.7)	2670 (37.8)	749 (37.6)	
Prince Edward Island	31 (0.3)	27 (0.4)	4 (0.2)	
Quebec	1829 (20.2)	1313 (18.6)	516 (25.9)	
Saskatchewan	296 (3.3)	233 (3.3)	63 (3.2)	
Territories [b]	26 (0.3)	26 (0.4)	0 (0.0)	
Ethnicity [c]				.961
Indigenous	272 (3.1)	213 (3.1)	59 (3.1)	
Not Indigenous	8425 (96.9)	6587 (96.9)	1838 (96.9)	
Experienced suicidal thoughts or feelings in the previous 2 weeks				<.001
Yes	599 (6.7)	433 (6.2)	166 (8.4)	
No	8371 (93.3)	6569 (93.8)	1802 (91.6)	

[a] Includes transgender woman, transgender man, and non-binary. [b] Includes Northwest Territories, Nunavut, and Yukon. [c] Participants were asked, "What is your family ethnicity" and were able to select multiple options. Respondents were classified as Indigenous if they self-identified having a family of Indigenous origins, even if they identified additional ethnic categories.

Supplementary Table 2: Comparison of respondents who did and did not select "Prefer not to say" to the question "Have you done or experienced any of the following, as a result of the COVID-19 pandemic in the past two weeks?" (n, row %)

	**Overall study sample**	**Experienced suicidal thoughts or feelings in the previous 2 weeks: Prefer not to say**		
	**(n, column %)** (n=7041 [a])	No (n=7002, 99.1%)	Yes (n=66, 0.9%)	Chi-square test p-value
Gender				.043
Cisgender man	3385 (48.1)	3350 (48.0)	35 (53.8)	
Cisgender woman	3605 (51.2)	3577 (51.3)	28 (43.1)	
Non-cisgender [b]	51 (0.7)	49 (0.7)	2 (3.1)	
Age				.059
18-34 years	884 (12.6)	871 (12.5)	13 (20.0)	
35-64 years	3967 (56.3)	3928 (56.3)	39 (60.0)	
65+ years	2190 (31.1)	2177 (31.2)	13 (20.0)	
Household income				.015
Under $25k	509 (7.3)	498 (7.2)	11 (17.7)	
$25k=<$50k	1266 (18.2)	1254 (18.2)	12 (19.4)	
$50k=<$100k	2465 (35.5)	2447 (35.6)	18 (29.0)	
$100k +	2703 (38.9)	2682 (39.0)	21 (33.9)	
Province				.874
Alberta	857 (12.2)	848 (12.2)	9 (13.8)	
British Columbia	1049 (14.9)	1042 (14.9)	7 (10.8)	
Manitoba	266 (3.8)	262 (3.8)	4 (6.2)	
New Brunswick	148 (2.1)	146 (2.1)	2 (3.1)	
Newfoundland and Labrador	103 (1.5)	103 (1.5)	0 (0.0)	
Nova Scotia	363 (5.2)	358 (5.1)	5 (7.7)	
Ontario	2659 (37.8)	2633 (37.7)	26 (40.0)	
Prince Edward Island	27 (0.4)	27 (0.4)	0 (0.0)	
Quebec	1311 (18.6)	1300 (18.6)	11 (16.9)	
Saskatchewan	233 (3.3)	232 (3.3)	1 (1.5)	
Territories [c]	25 (0.4)	25 (0.4)	0 (0.0)	
Ethnicity [d]				.107
Indigenous	209 (3.1)	205 (3.1)	4 (6.7)	
Not Indigenous	6569 (96.9)	6513 (96.9)	56 (93.3)	

[a] Final sample is composed of 7068 participants who participated in only one survey round; sensitivity analysis excludes 27 participants who selected "Two-Spirit", "Not listed", or "Prefer not to answer" in the gender question. [b] Includes transgender woman, transgender man, and non-binary. [c] Includes Northwest Territories, Nunavut, and Yukon. [d] Participants were asked, "What is your family ethnicity" and were able to select multiple options. Respondents were classified as Indigenous if they self-identified having a family of Indigenous origins, even if they identified additional ethnic categories.

Supplementary Table 3a: Results of logistic regression models for reporting having experienced COVID-related suicidal thoughts or feelings in the previous 2 weeks ("Prefer not to say" as "yes")– Sample characteristics (odds ratio, 95% confidence interval) (n=7041 [a])

	Unadjusted models	Adjusted model (including all sociodemographic factors and study round)
Gender		
Cisgender man	1.05 (0.87, 1.27)	1.32 (1.07, 1.64)
Cisgender woman	Reference	Reference
Non-cisgender [b]	8.36 (4.67, 14.97)	3.37 (1.45, 7.82)
Age		
18-34 years	5.64 (4.10, 7.75)	2.78 (1.90, 4.07)
35-64 years	2.95 (2.23, 3.90)	2.06 (1.52, 2.80)
65+ years	Reference	Reference
Household income		
Under $25k	2.71 (2.02, 3.63)	1.29 (0.90, 1.85)
$25k=<$50k	1.30 (1.00, 1.69)	0.99 (0.72, 1.34)
$50k=<$100k	1.05 (0.83, 1.31)	0.84 (0.65, 1.09)
$100k +	Reference	Reference
Province		
Alberta	1.06 (0.79, 1.43)	0.98 (0.71, 1.35)
British Columbia	0.88 (0.65, 1.18)	0.99 (0.73, 1.35)
Manitoba	0.78 (0.44, 1.36)	0.87 (0.49, 1.52)
New Brunswick	0.39 (0.14, 1.05)	0.55 (0.22, 1.38)
Newfoundland and Labrador	1.00 (0.46, 2.19)	1.36 (0.59, 3.13)
Nova Scotia	0.98 (0.63, 1.53)	0.92 (0.59, 1.44)
Ontario	Reference	Reference
Prince Edward Island	0.53 (0.07, 3.92)	0.46 (0.06, 3.64)
Quebec	0.63 (0.46, 0.86)	0.86 (0.62, 1.18)
Saskatchewan	1.37 (0.85, 2.20)	1.12 (0.66, 1.91)
Territories [c]	1.15 (0.27, 4.89)	1.22 (0.26, 5.79)
Marital status		
Single	2.60 (2.12, 3.19)	1.70 (1.31, 2.20)
Married or partnered	Reference	Reference
Separated, divorced, widowed	1.37 (1.04, 1.80)	1.41 (1.02, 1.94)
LGBT2Q+: Yes or unsure [d]	3.39 (2.63, 4.35)	1.60 (1.19, 2.16)
Ethnicity: Indigenous [e]	2.89 (1.98, 4.20)	1.85 (1.21, 2.83)
Pre-existing mental health condition: Yes [f]	6.41 (5.28, 7.77)	5.05 (4.07, 6.27)
Study round		
Round 1 (May 2020)	Reference	Reference
Round 2 (September 2020)	1.19 (0.97, 1.46)	1.44 (1.14, 1.82)

Round 3 (January 2021)	0.79 (0.61, 1.03)	1.01 (0.76, 1.35)

[a] Final sample is composed of 7068 participants who participated in only one survey round; sensitivity analyses exclude 27 participants who selected “Two-Spirit”, “Not listed”, or “Prefer not to answer” in the gender question. [b] Includes transgender woman, transgender man, and non-binary. [c] Includes Northwest Territories, Nunavut, and Yukon. [d] Participants were asked, “Do you identify as being LGBT2Q+ (lesbian, gay, bisexual, trans, two-spirit, queer, etc.)?” [e] Participants were asked, "What is your family ethnicity" and were able to select multiple options. Respondents were classified as Indigenous if they self-identified having a family of Indigenous origins, even if they identified additional ethnic categories. [f] Participants were asked, “Do you identify as a person who has a pre-existing (prior to COVID-19) mental health condition?”

Supplementary Table 3b: Results of logistic regression models for reporting having experienced COVID-related suicidal thoughts or feelings in the previous 2 weeks (“Prefer not to say” as “no”)– Sample characteristics (odds ratio, 95% confidence interval) (n=7041 [a])

	Unadjusted models	Adjusted model (including all sociodemographic factors and study round)
Gender		
Cisgender man	1.01 (0.83, 1.23)	1.27 (1.01, 1.59)
Cisgender woman	Reference	Reference
Non-cisgender [b]	8.04 (4.42, 14.63)	2.59 (1.06, 6.30)
Age		
18-34 years	6.34 (4.47, 8.99)	3.21 (2.12, 4.85)
35-64 years	3.26 (2.39, 4.45)	2.34 (1.67, 3.28)
65+ years	Reference	Reference
Household income		
Under $25k	2.61 (1.91, 3.57)	1.28 (0.87, 1.89)
$25k=<$50k	1.31 (0.99, 1.73)	1.04 (0.75, 1.44)
$50k=<$100k	1.06 (0.83, 1.35)	0.89 (0.68, 1.17)
$100k +	Reference	Reference
Province		
Alberta	1.06 (0.79, 1.43)	0.96 (0.68, 1.35)
British Columbia	0.88 (0.65, 1.18)	1.03 (0.75, 1.43)
Manitoba	0.78 (0.44, 1.36)	0.78 (0.42, 1.45)
New Brunswick	0.39 (0.14, 1.05)	0.49 (0.17, 1.37)
Newfoundland and Labrador	1.00 (0.46, 2.19)	1.61 (0.70, 3.71)
Nova Scotia	0.98 (0.63, 1.53)	0.82 (0.50, 1.33)
Ontario	Reference	Reference
Prince Edward Island	0.53 (0.07, 3.92)	0.52 (0.07, 4.16)
Quebec	0.63 (0.46, 0.86)	0.86 (0.61, 1.21)
Saskatchewan	1.37 (0.85, 2.20)	1.20 (0.70, 2.08)
Territories [c]	1.15 (0.27, 4.89)	1.49 (0.31, 7.17)

Marital status		
Single	2.51 (2.02, 3.12)	1.64 (1.25, 2.15)
Married or partnered	Reference	Reference
Separated, divorced, widowed	1.28 (0.95, 1.72)	1.31 (0.93, 1.85)
LGBT2Q+: Yes or unsure [d]	3.60 (2.78, 4.68)	1.66 (1.22, 2.27)
Ethnicity: Indigenous [e]	2.90 (1.95, 4.31)	1.86 (1.20, 2.89)
Pre-existing mental health condition: Yes [f]	6.75 (5.50, 8.29)	5.24 (4.16, 6.59)
Study round		
Round 1 (May 2020)	Reference	Reference
Round 2 (September 2020)	1.27 (1.03, 1.58)	1.55 (1.21, 1.98)
Round 3 (January 2021)	0.78 (0.59, 1.03)	1.03 (0.76, 1.41)

[a] Final sample is composed of 7068 participants who participated in only one survey round; sensitivity analyses exclude 27 participants who selected "Two-Spirit", "Not listed", or "Prefer not to answer" in the gender question. [b] Includes transgender woman, transgender man, and non-binary. [c] Includes Northwest Territories, Nunavut, and Yukon. [d] Participants were asked, "Do you identify as being LGBT2Q+ (lesbian, gay, bisexual, trans, two-spirit, queer, etc.)?" [e] Participants were asked, "What is your family ethnicity" and were able to select multiple options. Respondents were classified as Indigenous if they self-identified having a family of Indigenous origins, even if they identified additional ethnic categories. [f] Participants were asked, "Do you identify as a person who has a pre-existing (prior to COVID-19) mental health condition?"

Supplementary Table 4a: Results of logistic regression models for reporting having experienced COVID-related suicidal thoughts or feelings in the previous 2 weeks ("Prefer not to say" as "yes") – Pandemic-related stressors (odds ratio, 95% confidence interval) (n=7041 [a])

	Unadjusted models	Adjusted models (individual models for each stressor, also including sociodemographic factors and study round)
Financial stressors		
Financial concerns	3.45 (2.85, 4.17)	2.43 (1.96, 3.02)
Losing their job	3.07 (2.54, 3.72)	2.43 (1.95, 3.03)
Relationship stressors		
Experiencing relationship challenges	3.18 (2.62, 3.86)	2.90 (2.30, 3.65)
Being safe from physical or emotional domestic violence	2.51 (1.95, 3.23)	1.99 (1.46, 2.70)
Increased substance use		
Drinking alcohol	2.24 (1.83, 2.75)	1.92 (1.52, 2.43)
Use of tobacco products	4.46 (3.44, 5.79)	2.55 (1.85, 3.51)
Use of cannabis products	5.25 (4.15, 6.64)	2.86 (2.16, 3.78)
Use of prescribed medication	7.66 (5.86, 10.00)	3.41 (2.45, 4.75)
Use of other psychoactive substances	10.23 (6.68, 15.69)	4.06 (2.31, 7.13)
COVID-19 stressors		

Tested positive for COVID-19	11.16 (6.23, 19.98)	4.21 (1.79, 9.88)
Someone in their household tested positive for COVID-19	6.62 (3.62, 12.12)	1.63 (0.60, 4.46)
Family member/loved one living at a different address tested positive for COVID-19	1.27 (0.84, 1.92)	1.06 (0.65, 1.75)
Fear of getting severely sick or dying	1.88 (1.57, 2.26)	1.61 (1.31, 1.98)

[a] Final sample is composed of 7068 participants who participated in only one survey round; sensitivity analyses exclude 27 participants who selected “Two-Spirit”, “Not listed”, or “Prefer not to answer” in the gender question.

Supplementary Table 4b: Results of logistic regression models for reporting having experienced COVID-related suicidal thoughts or feelings in the previous 2 weeks (“Prefer not to say” as “no”) – Pandemic-related stressors (odds ratio, 95% confidence interval) (n=7041 [a])

	Unadjusted models	Adjusted models (individual models for each stressor, also including sociodemographic factors and study round)
Financial stressors		
Financial concerns	3.61 (2.95, 4.43)	2.47 (1.96, 3.11)
Losing their job	3.36 (2.74, 4.10)	2.60 (2.06, 3.28)
Relationship stressors		
Experiencing relationship challenges	3.28 (2.67, 4.03)	2.84 (2.23, 3.62)
Being safe from physical or emotional domestic violence	2.52 (1.93, 3.29)	2.04 (1.48, 2.80)
Increased substance use		
Drinking alcohol	2.38 (1.92, 2.94)	1.96 (1.53, 2.50)
Use of tobacco products	4.71 (3.60, 6.18)	2.57 (1.84, 3.57)
Use of cannabis products	5.49 (4.30, 7.02)	2.89 (2.17, 3.86)
Use of prescribed medication	7.75 (5.88, 10.22)	3.39 (2.41, 4.78)
Use of other psychoactive substances	10.95 (7.11, 16.87)	4.35 (2.46, 7.70)
COVID-19 stressors		
Tested positive for COVID-19	13.07 (7.29, 23.43)	4.97 (2.11, 11.68)
Someone in their household tested positive for COVID-19	7.74 (4.23, 14.18)	1.93 (0.71, 5.26)
Family member/loved one living at a different address tested positive for COVID-19	1.42 (0.93, 2.17)	1.16 (0.70, 1.93)
Fear of getting severely sick or dying	1.98 (1.62, 2.40)	1.66 (1.33, 2.07)

[a] Final sample is composed of 7068 participants who participated in only one survey round; sensitivity analyses exclude 27 participants who selected “Two-Spirit”, “Not listed”, or “Prefer not to answer” in the gender question.
